# Acute myeloid leukemia cells and MSC-derived exosomes inhibiting transformation in myelodysplastic syndrome

**DOI:** 10.1007/s12672-023-00714-2

**Published:** 2023-06-29

**Authors:** Xiaoli Liu, Fanggang Ren, Shuo Li, Na Zhang, Jeffrey J. Pu, Hongyu Zhang, Zhifang Xu, Yanhong Tan, Xiuhua Chen, Jianmei Chang, Hongwei Wang

**Affiliations:** 1 Shanxi Medical University, 56 Xinjian South Road, Taiyuan, 030001 People’s Republic of China; 2 Laboratory of Hematology, Second Hospital of Shanxi Medical University, Taiyuan, China; 3 The Key Laboratory of Molecular Diagnosis and Treatment of Hematological Diseases of Shanxi Province, 382 Wuyi Road, Taiyuan, 030001 People’s Republic of China; 4 Department of Medical Laboratory, Second Hospital of Shanxi Medical University, Taiyuan, China; 5 Department of Medicine, University of Arizona College of Medicine, Tucson, AZ USA

**Keywords:** MDS, AML, Mesenchymal stem cells, Transformation, Exosomes

## Abstract

**Aims:**

To investigate the mechanism of exosomes' role in the transformation of MDS to AML.

**Methods:**

Exosomes in culture supernatants of MDS and AML cell lines, were extracted by ultrafiltration and identified in three ways: morphology, size, and exosome protein surface markers. Exosomes from AML cell lines were then co-cultured with MDS cell lines and their impacts on MDS cell microenvironment, proliferation, differentiation, cell cycle, and apoptosis were analyzed by CCK-8 assay and flow cytometry. Furthermore, exosomes from MSC were extracted for further authentication.

**Results:**

The transmission electron microscopy, nanoparticle tracking analysis, Western blotting, and flow cytometry methods all verify that ultrafiltration is a reliable method to extract exosomes in the culture medium. Exosomes from AML cell lines inhibit the proliferation of MDS cell lines, block cell cycle progression, and promote apoptosis and cell differentiation. It also leads to increased secretion of tumor necrosis factor-α (TNF-α) and reactive oxygen species (ROS) in MDS cell lines. In addition, MSC-derived exosomes were found to inhibit the proliferation of MDS cell lines, arrest cell cycle progression, promote apoptosis, and inhibit differentiation.

**Conclusion:**

Ultrafiltration is a proper methodology in extracting exosomes. The exosomes of AML origin and MSC origin may play a role in MDS leukemia transformation via targeting TNF-α/ROS-Caspase3 pathway.

## Background

Myelodysplastic syndromes (MDS) are a group of clonal hematopoietic stem cell disorders that are characterized by inefficient hematopoiesis, hemocytopenia, and an increase in blast cells, with nearly one-third of patients at risk of progression into acute myeloid leukemia(AML) [1, 2]. In the latest WHO classification (2022 edition), MDS-IB was divided into MDS-IB1 and MDS-IB2 according to the number of primitive cells, and these patients are easy to transform into AML. In addition, the distinction between MDS and AML has been weakened to the point that MDS-IB2 is considered AML in some cases [3–5]. The transition from MDS to AML has been studied for many years and the mutated genes associated with AML have previously been divided into two categories: those that activate growth factor signaling to promote cell proliferation and transcription factors that affect hematopoietic cell differentiation and immature cell numbers, leading to MDS, in the case of both types of mutation, to the possibility of secondary AML [6–8]. However, later studies have revealed that the mechanisms of MDS to AML conversion are much more complex than this two-category approach, such as clonal evolution, chromosomal abnormalities, epigenetic changes, immunosuppression, and selective splicing [9].

Exosomes are extracellular vesicles 30–150 nm in diameter formed by cells by way of endocytosis [10]. Exosomes contain proteins, mRNA, miRNA, enzymes, transcription factors, molecular partners and signaling molecules, etc. Almost all cells can produce exosomes and they are also widely found in plasma, urine, cerebrospinal fluid, and other body fluids [11]. Exosomes can be absorbed by nearby and distant cells, thus enabling the transfer of genetic material and signaling proteins between cells, playing a role in cell development, functional integrity, and the progression of hematological malignancies [12]. Its role in mediating intercellular communication is mainly through the direct fusion of the exosome membrane with the cytolemma to release its contents, or the membrane proteins of the exosome can be sheared by proteases and the sheared fragments can act as ligands to bind to receptors on the cell membrane, thereby activating intracellular signaling pathways [13]. The tumor microenvironment (TME) mainly consists of tumor cells, extracellular matrix, and tumor regulators. Tumor cell-derived exosomes can modify the TME by affecting signaling pathways such as apoptosis, proliferation, differentiation, invasion, and angiogenesis, ultimately affecting the growth, invasion, and metastasis of tumor cells [14–16].

Apoptosis is a programmed cell death caused by the death receptor pathway or the mitochondrial pathway [17]. Tumor necrosis factor-α (TNF-α) is a small molecule protein primarily secreted by monocytes and macrophages, that can kill tumor cells and cause apoptosis [18]. Reactive oxygen species (ROS) are certain metabolites of oxygen and some oxygen-containing products of reactions, which are produced and increased by mitochondria when cells are stimulated, and elevated ROS can lead to apoptosis by activating caspases [19, 20]. Both apoptotic pathways ultimately focus on the activation of the Caspase family of proteases, in the end, leading to DNA fragmentation, chromatin condensation, cellular crumpling, and membrane bubbles [21].

At present, there is still no effective therapeutic methodology in treating MDS. The low and intermediate risk MDS is mainly managed via hypomethylating agents and supportive care, such as transfusion therapy. Cytotoxic chemotherapy, targeted therapy, and immunomodulatory therapy are the common managements for high-risk MDS, Allogeneic hematopoietic stem cell transplantation (HSPC) is the only treatment possible to cure MDS, but the treatment-related mortality risk and the source of stem cells are still the biggest obstacles [22, 23].

It is now generally accepted that tumor cell-derived exosomes can promote disease progression and metastasis [24]. In this study, we first report that exosomes from AML cell lines could inhibit the transformation of MDS by promoting apoptosis of MDS cell lines through the TNF-α/ROS-Caspase3 pathway. In addition, MSC-derived exosomes could inhibit the proliferation of MDS cell lines. These observation will help us to further understand the mechanisms of MDS progression and leukemic transformation.

## Methods

### Cell culture

The human myelodysplastic syndrome cell lines SKM-1 and MUTZ-1 were purchased from Otwo Biotech (Shenzhen, China), and the human myeloid leukemia cell lines THP-1, K562, and HL-60 are routinely maintained in our laboratory. SKM-1 was cultured in IMDM with 10% FBS and 1% penicillin, MUTZ-1, THP-1, K562 and HL-60 were cultured in RPMI-1640 with 10% FBS and 1% penicillin, and all cell lines were cultured at 37 °C and 5% CO_2_.

### Exosome extraction

When density reached 60–70%, cells were collected by centrifugation at 300 g, 4 °C, 10 min, the supernatant was collected after 48 h of further incubation in a serum-free medium. The supernatant was centrifuged at 300 g, 4 °C, 10 min to remove cellular components; 2000 g, 4 °C, 10 min to remove dead cells; then 10000 g,4 °C,30 min to remove cell fragments. The supernatant was filtrated via a 0.22 μm filter to remove apoptotic vesicles. The exosomes were collected by ultrafiltration using a 10 KD ultrafiltration tube (Millipore, USA). Finally, the ultrafiltrate was resuspended in PBS to elute the medium components.

### Exosome identification

Transmission electron microscope (TEM) and Nanoparticle Tracking Analysis (NTA) were sent to Beijing Ovison Gene Technology Company for analysis. Exosome proteins were lysed using RIPA lysate (Thermo Scientific, USA). Rough quantification was performed using a BCA protein concentration assay kit (SEVEN, Beijing, China). Equal amounts of exosome protein (60 μg) were subjected to polyacrylamide gel electrophoresis (SDS-PAGE), followed by transferring the protein onto 0.45 μm PVDF membranes (Roche, UK). Using 5% skimmed milk powder for closure, incubated with primary antibody CD63, TSG101, Calnexin for 1.5 h and HRP-labelled goat anti-rabbit secondary antibody for 2 h at room temperature in a shaker. The results were finally developed using the ECL (SEVEN, Beijing, China) chemiluminescence method. Exosomes were identified by flow cytometry method using CD9 -FITC, CD63- PE, CD81- FITC, CD33- APC, and CD14- APC antibodies as the surface markers.

### Exosome uptake by MDS cell lines

PKH26 (MCE, China) binds to the lipid region of the cell membrane, showing as a sign of red fluorescence. The labeled exosomes were co-cultured with the MDS cell line for 48 h and then observed under a fluorescent microscope and photographed for documentation.

### Flow cytometry

AML cell line-derived exosomes were co-cultured with MDS cell lines for 48 h before flow cytometric analyses of cell cycle, apoptosis, differentiation, cytokines, and ROS. Cells were fixed in pre-cooled 70% ethanol at 4 °C, stained using propidium iodide (PI) according to the Cell Cycle Assay Kit (Bioss, Beijing, China), and DNA content was determined by flow cytometry analysis, and the percentage of cells in G0/G1, S, and G2/M phases were counted for comparison. Apoptosis staining was performed using the Apoptosis Assay Kit (Bioss, Beijing, China). 10^5^ cells were taken in a flow tube, 5 μl FITC, and 5 μl PI were added, mixed, and incubated for 15 min at room temperature and protected from light and then tested on the machine. Cell differentiation and proliferation indicators were detected using CD11b-PE and CD117- PC5 antibodies. The Seven-item Cytokine Assay Kit (Uni-medica, Shenzhen, China) was used to detect cytokines in the culture supernatant. Detection of intracellular reactive oxygen levels were used the Reactive Oxygen Species Assay Kit (Beyotime, Shanghai, China), Briefly, the cells were resuspended in DCFH-D diluted with serum-free medium, incubated in a 37 °C cell incubator for 20 min, washed with serum-free medium and then assayed on the machine.

### Cell proliferation assays

Cell proliferation was measured using the Cell Counting Kit (CCK-8) (YEASEN, Shanghai, China). AML cell line-derived exosomes were co-cultured with the MDS cell line for 48 h and then inoculated into 96-well plates at 5 × 10^3^/well with 100 μ cell suspension per well, incubate at 37 °C, 5% CO_2_. The plate was removed at 0 d, 1 d, 2 d, 3 d, and 4 d, and 10 μl of CCK-8 reagent was added to each well and the culture was continued for 2 h. The absorbance (OD) values of the corresponding wells were measured at 450 nm and the cell proliferation curves were plotted.

### Western blotting

Proteins from MDS cell lines were extracted using RIPA lysate (Thermo Scientific, USA) after 48 h co-culturing The extracted proteins were quantified using the BCA protein concentration assay kit (SEVEN, Beijing, China). Equal amounts of protein (60 μg) were subjected to SDS-PAGE and subsequently transferred onto 0.45 μm PVDF membranes (Roche, UK). The primary antibody Caspase3 and β-actin were incubated for 1.5 h on a shaker at room temperature. Then the HRP-labelled secondary antibody was incubated for 2 h. The final development was performed by chemiluminescence using ECL (SEVEN, Beijing, China).

### Data analysis

SPSS (version 17.0) and GraphPad (Prism 7.0) were used for statistical processing and plotting. All data were obtained from three independent experiments and the means of the two samples were compared using the *t-test*. *p* < 0.05 was considered statistically significant.

## Results

### Identification of exosomes

**Fig. 1:**
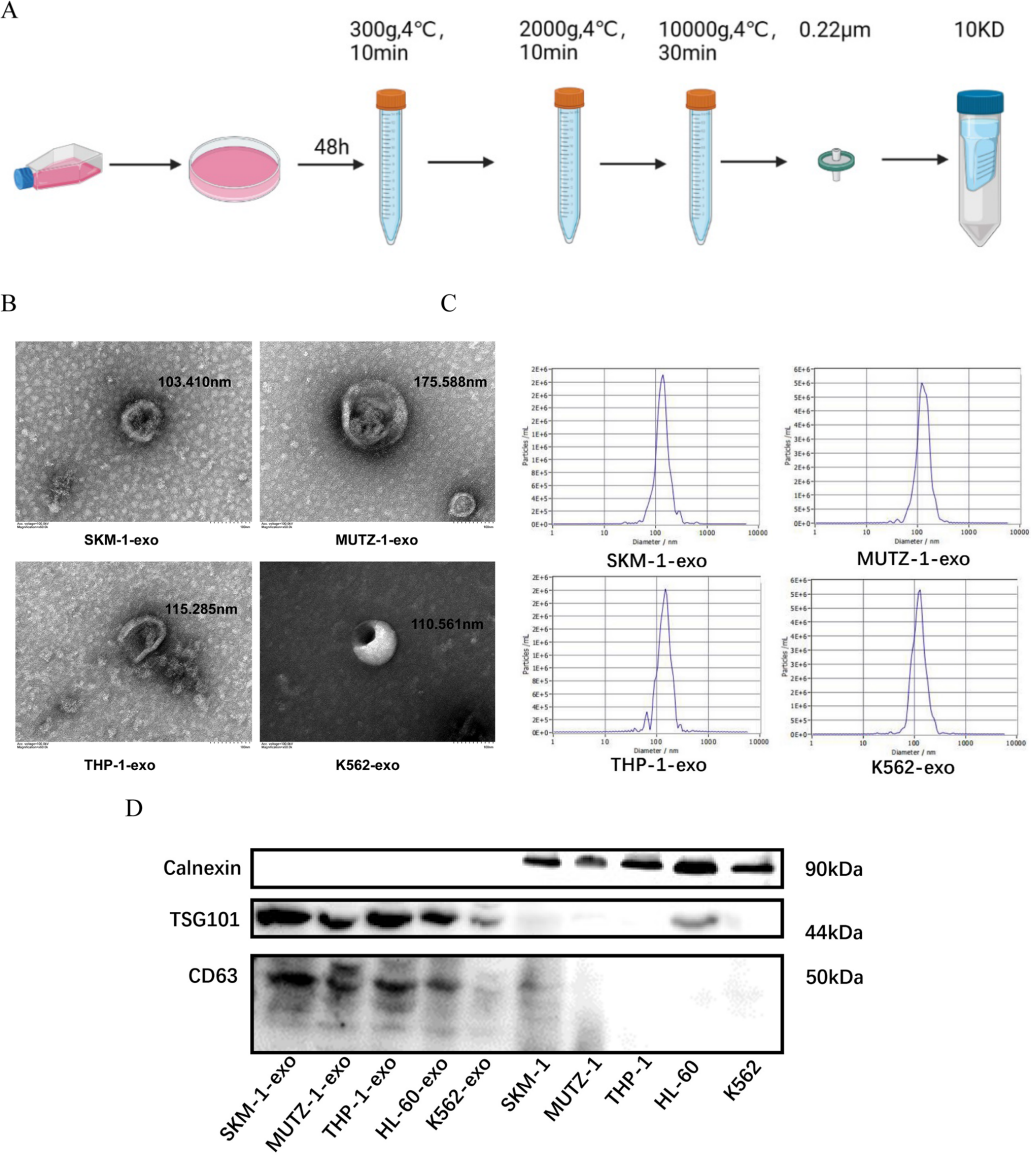

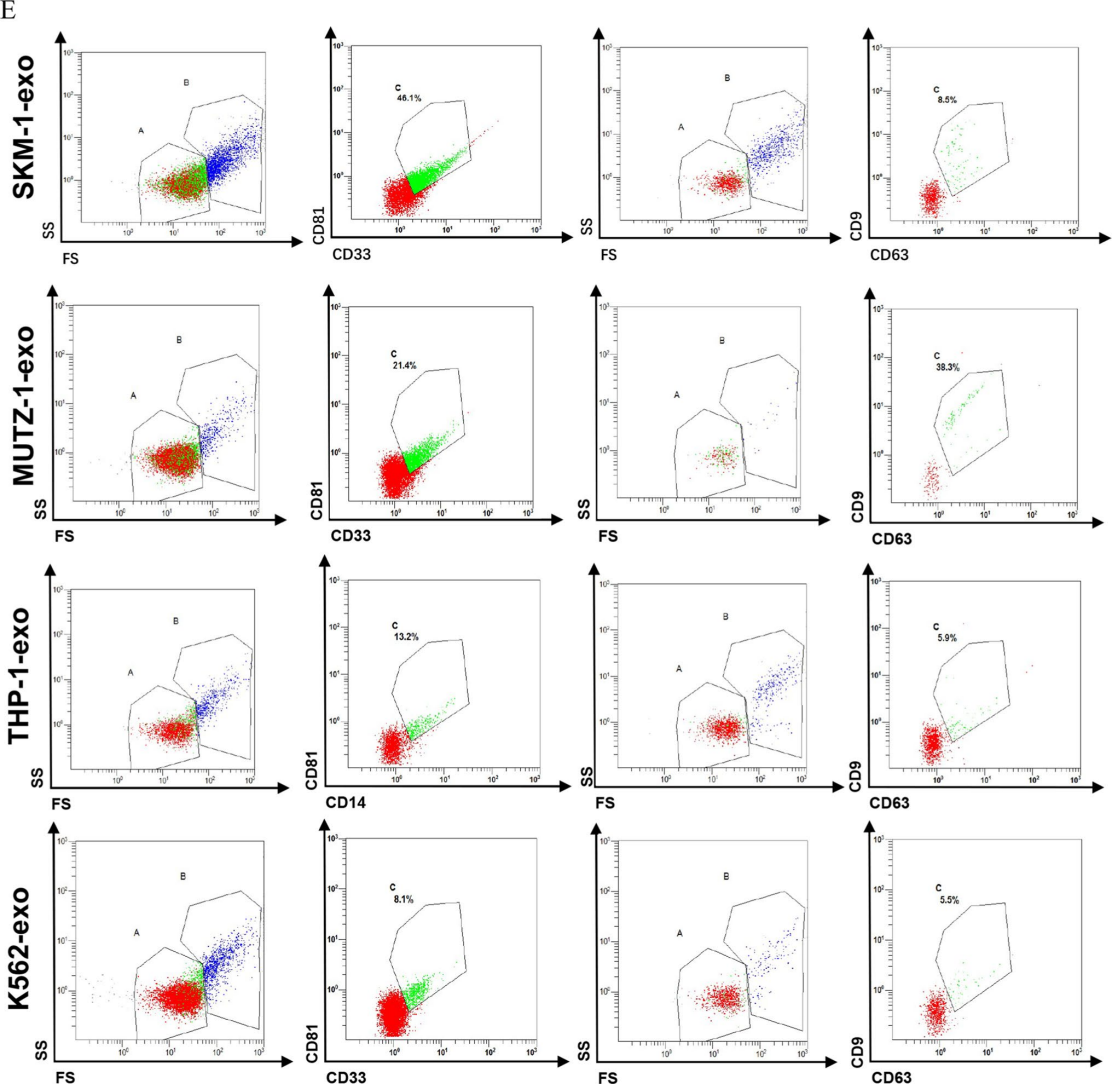
Identification of exosomes.** A**. Schematic diagram for exosome extraction by ultrafiltration. **B**. Morphological characteristics of exosomes under transmission electron microscopy. **C**. Results of nanoparticle tracking analysis chart with histograms indicating the distribution of particle sizes. **D**. Western blotting examine the expression of Calnexin which was used as negative control, TSG101 and CD63 which were considered as specific biomarkers on exosomes but cell lysis products. **E**. Flow cytometry detection of exosome signature proteins

Exosomes as described above were scanned by transmission electron microscopy, the exosomes were membrane vesicles less than 200 nm in diameter and resembling a teacup tray-like shape (Fig. 1A, B). Nanoparticle tracking analysis showed that the average direction of the exosomes was between 30 and 150 nm (Fig. 1C). Western blotting analysis showed that when exosomes highly expressed CD63 and TSG101 (positive makers for exosomes [25]), the cells expressed in low or absent; while exosomes were low in Calnexin expression (negative makers for exosomes [25]), the cells were high in its expression (Fig. 1D). The expression of protein markers such as CD81, CD63, and CD9 on the exosome surfaces was detected by flow cytometry analyses (Fig. 1E). The exosomes extracted by ultrafiltration met the requirements of the International Society for Extracellular Vesicles (ISEV) 2018 edition of Guidance Requirements for exosomes by morphology, particle size, and exosome surface protein expression analysis [26]. The extracted exosomes were used for subsequent experiments.

### AML cell line-derived exosomes can transfer into MDS cell lines

**Fig. 2:**
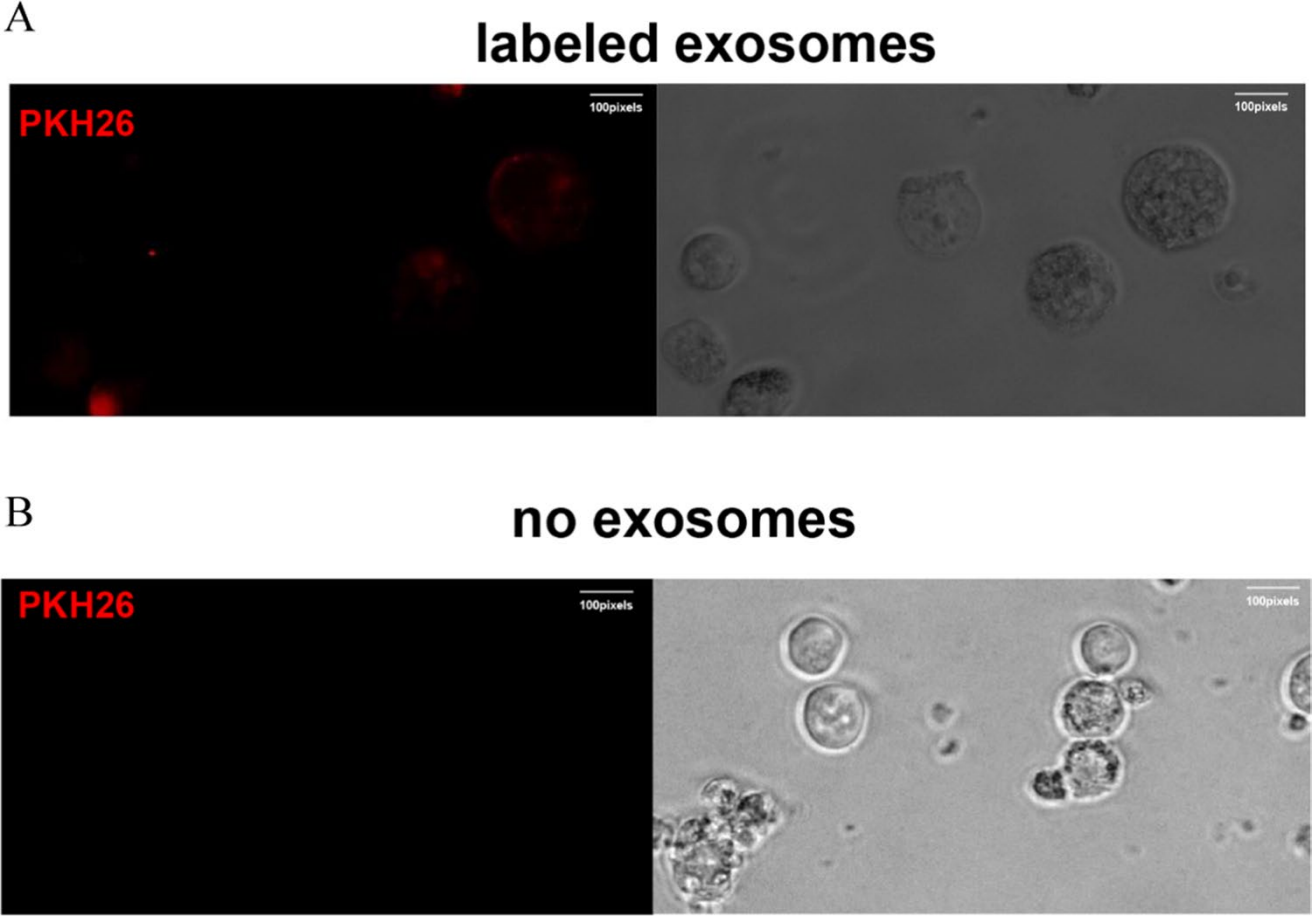
AML cell line-derived exosomes can transfer into MDS cell lines. **A**. Representative images of MDS cell line uptake of PKH26 labeled exosomes 48 h later. **B**. Image of PKH26 labeling without exosomes. Scale bar: 100 pixels

To identify whether exosomes of AML cell line could be translocated into the MDS cell line, the exosomes labeled with PKH26 were cultured with the MDS cell line. The result showed that the MDS cell line can take up the PKH26-labelled exosomes after two days of incubation (Fig. 2).

### AML cell line-derived exosomes inhibit proliferation and promote differentiation of MDS cell lines

**Fig. 3:**
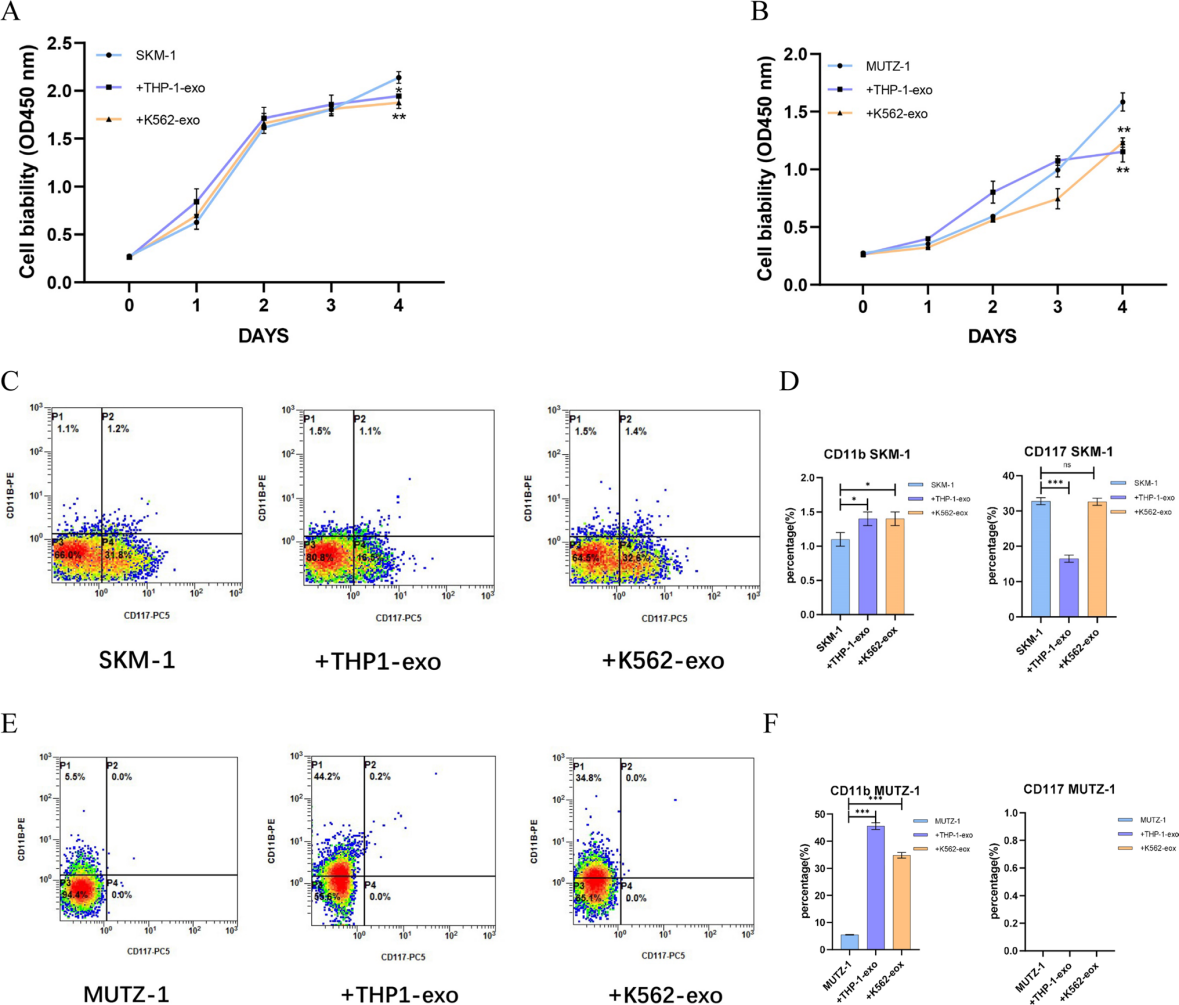
AML cell line-derived exosomes inhibit proliferation and promote differentiation of MDS cell lines. **A** and **B**. AML cell line-derived exosomes inhibit the proliferation of MDS cell lines. **C** and **D**. AML cell line-derived exosomes promote differentiation of SKM-1 cell lines. **E** and **F**. AML cell line-derived exosomes promote differentiation of MUTZ-1 cell lines. (*p < 0.05, **p < 0.01, ***p < 0.001)

After co-culture with 100 μg/ml THP-1-exo and K562-exo with SKM-1 and MUTZ-1 cells respectively, cell proliferation was detected by CCK-8 assay. Compared with the control group, THP-1-exo, and K562-exo resulted in a significant reduction in the growth potential of SKM-1 and MUTZ-1 on day 4 (Fig. 3A, B). It means that AML-exo has an inhibitory effect on MDS proliferation. Cell differentiation and proliferation markers were analyzed by flow cytometry. Compared to the control, THP-1-exo and K562-exo promoted the differentiation of SKM-1 cells, THP-1-exo inhibits the expression of the proliferation marker CD117 in SKM-1 cells, which is consistent with the results of CCK-8, however, K562-exo did not affect SKM-1 (Fig. 3C, D). Meanwhile, THP-1-exo and K562-exo promoted the differentiation of MUTZ-1 cells, and the cell proliferation marker CD117 was not detected in MUTZ-1 cells (Fig. 3E, F).

### AML cell line-derived exosomes arrest the progression of the MDS cell line cycle

**Fig. 4:**
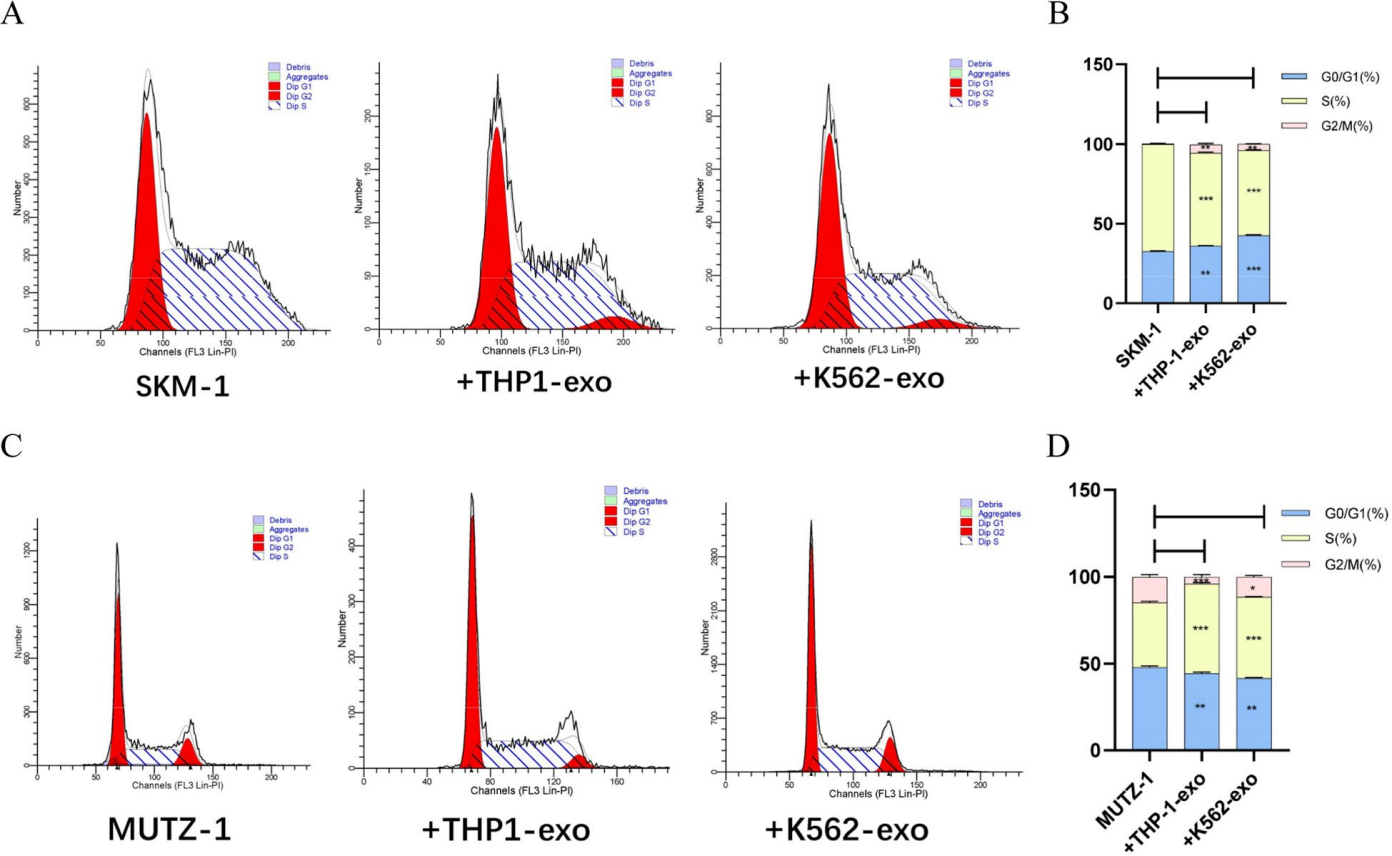
AML cell line-derived exosomes arrest the progression of the MDS cell line cycle. **A** and **B**. AML cell line-derived exosomes blocked SKM-1 cells in the G0/G1 phase. **C** and **D**. AML cell line-derived exosomes blocked MUTZ-1 cells in the S phase. (*p < 0.05, **p < 0.01, ***p < 0.001)

To investigate the regulation of AML-exo on the cell cycle of MDS cell lines, after co-culture with 100 μg/ml THP-1-exo and K562-exo for 48 h with SKM-1 and MUTZ-1 cells severally, cell cycle progression analysis by flow cytometry revealed that THP-1-exo caused SKM-1 cells to block in the G0/G1 phase (*p* < 0.01) and that K562-exo made SKM-1 cells to stop in the G0/G1 phase (*p* < 0.001) (Fig. 4A, B). THP-1-exo prevented MUTZ-1 cells from entering the S phase (*p* < 0.001) and K562-exo resulted in MUTZ-1 cells being blocked in the S phase (*p* < 0.001) (Fig. 4C, D).

### AML cell line-derived exosomes promote apoptosis in MDS cell lines

**Fig. 5:**
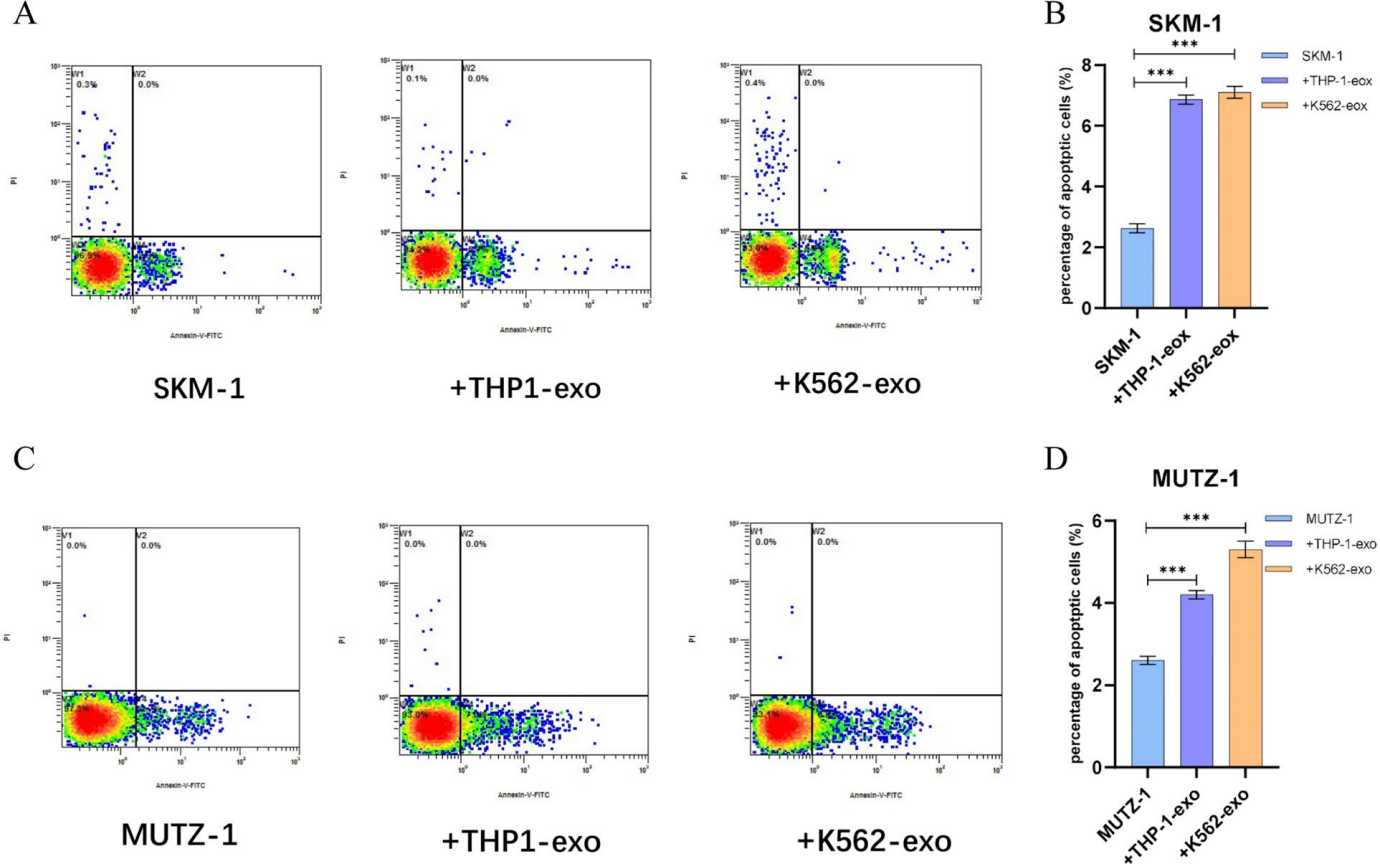
AML cell line-derived exosomes promote apoptosis in MDS cell lines. **A** and **B**. AML cell line-derived exosomes promote SKM-1 apoptosis. **C** and **D**. AML cell line-derived exosomes enhanced MUTZ-1 apoptosis. (*p < 0.05, **p < 0.01, ***p < 0.001)

After co-culturing 100 μg/ml THP-1-exo and K562-exo with SKM-1 and MUTZ-1 cells for 48 h separately, apoptosis was analyzed by flow cytometry analyses. THP-1-exo (*p* < 0.001) and K562-exo (*p* < 0.001) co-culturing increased SKM-1 cell apoptosis (Fig. 5A, B). Apoptosis was similarly enhanced in co-cultured MUTZ-1 cell (*p* < 0.001) (Fig. 5C, D).

### AML cell line-derived exosomes promote apoptosis in MDS cell lines via Caspase3

**Fig. 6:**
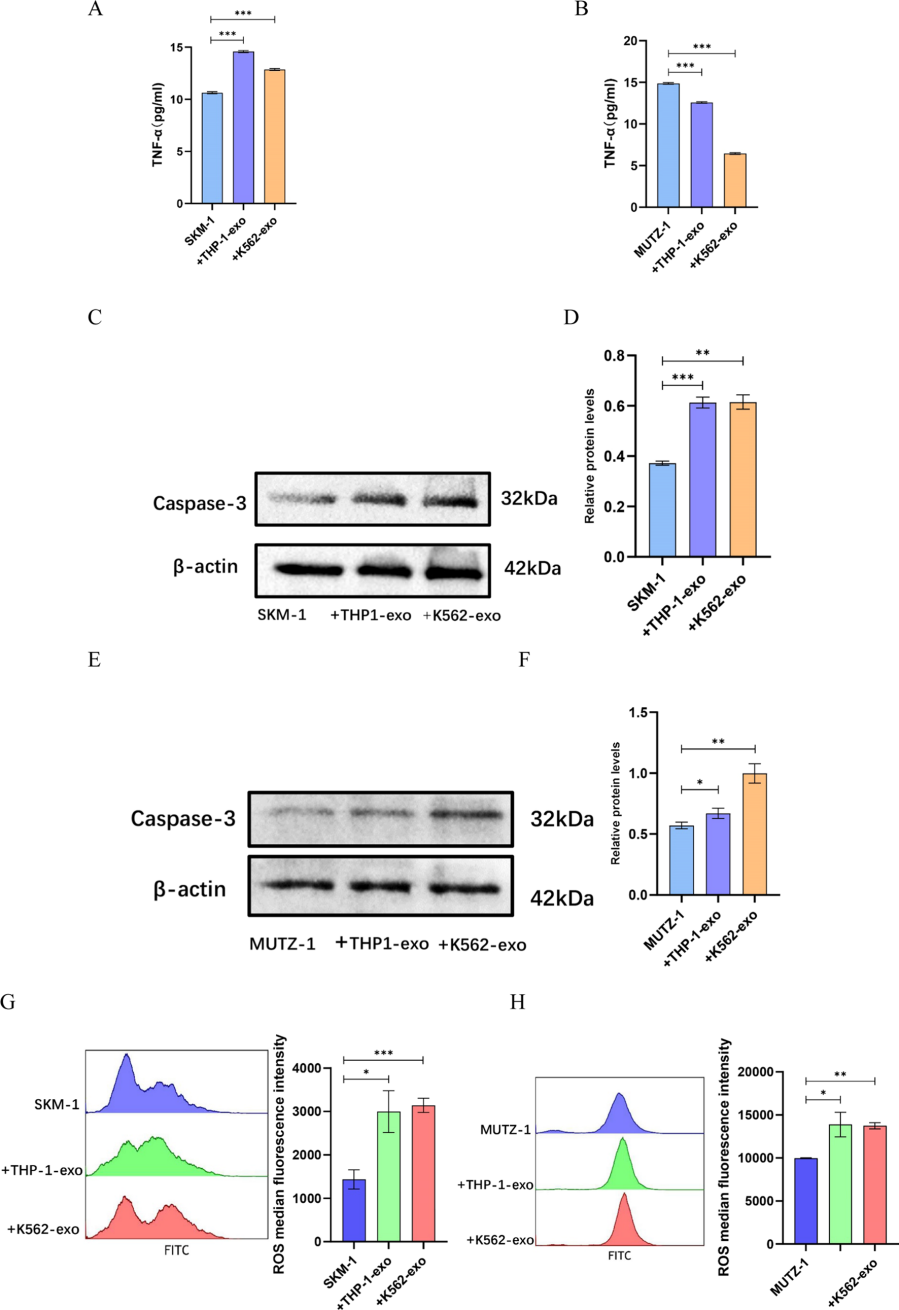
AML cell line-derived exosomes promote apoptosis in MDS cell lines via Caspase3.** A**. AML cell line-derived exosomes cause more TNF-α release from SKM-1. **B**. AML cell line-derived exosomes cause less TNF-α release from MUTZ-1. **C ~ F**. AML cell-derived exosomes increased Caspase3 release from MDS cells. **G** and **H**. AML cell-derived exosomes increased ROS release from MDS cells. (*p < 0.05, **p < 0.01, ***p < 0.001)

The effect of AML cell line-derived exosomes on the microenvironment was analyzed by flow cytometry. The results showed that AML cell line-derived exosomes increased the release of TNF-α from SKM-1 (p < 0.001) (Fig. 6A). In contrast, AML cell line-derived exosomes caused MUTZ-1 to release less TNF-α (p < 0.001) (Fig. 6B). The downstream associated protein Caspase3, a protein ultimately activated by multiple death receptor signaling pathways, was examined for how TNF-α increases apoptosis. It was found that exosomes of AML cell origin increased the release of Caspase3 from MDS cells (Fig. 6C ~ F). These data indicated that TNF-α activates its receptor TNF-R1 in SKM-1 cells, which ultimately activates Caspase3 leading to apoptosis; whereas, in MUTZ-1 cells there is an additional activator that elevates Caspase3 expression. It has been found that excess ROS can activate the mitochondrial apoptotic pathway, which induces apoptosis by releasing apoptosis-inducing factors to activate Caspase3. Exosomes of AML cell line origin were found to increase the release of reactive oxygen species from MUTZ-1 after 48 h of co-culture by flow cytometry (Fig. 6G, H).

### The effect of mesenchymal stem cell-derived exosomes on MDS cell lines

**Fig. 7:**
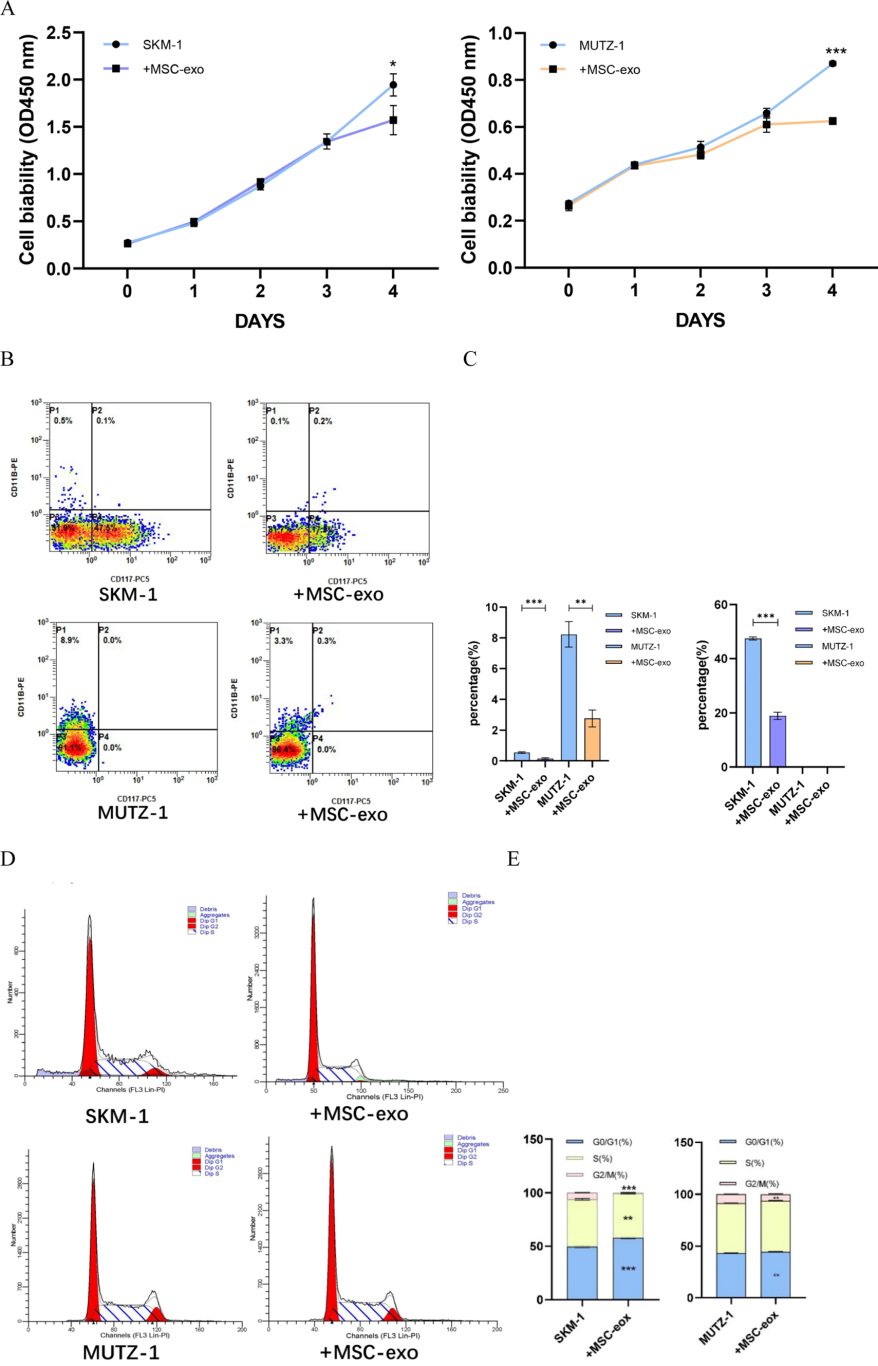

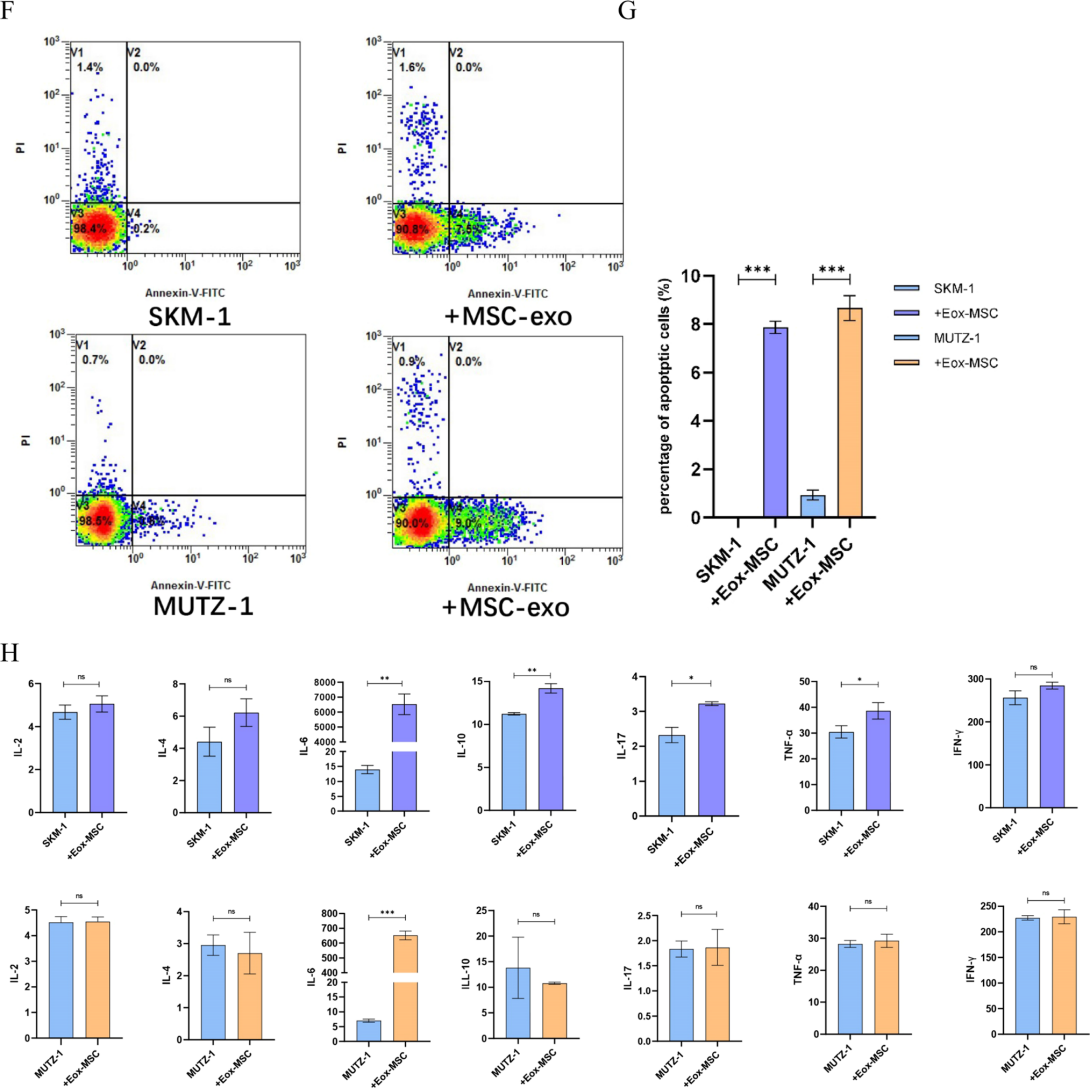
Effect of MSC-derived exosomes on MDS cell lines. **A**. MSC-derived exosomes inhibit the proliferation of MDS cell lines. **B** and **C**. MSC-derived exosomes inhibit the differentiation and proliferation of MDS cell lines. **D** and **E**. MSC-derived exosomes blocked MDS cell in the G0/G1 phase. **F** and **G**. MSC-derived exosomes promote apoptosis in MDS cells. **H**. MSC-derived exosomes elevate cytokine levels in some MDS cells. (*p < 0.05, **p < 0.01, ***p < 0.001)

To investigate the effect of MSC-derived exosomes on the biological function of MDS cell lines, after extracting MSC-derived exosomes by the above method and co-culturing with 100 μg/ml MSC-exo for 48 h with MDS cell lines respectively. Cell proliferation profile was analyzed by the CCK-8 method, and compared to the control group. MSC-exo significantly decreased the growth potential of MDS cells at day 4 (*p* < 0.05, *p* < 0.001) (Fig. 7A), which indicates that MSC-derived exosomes inhibited the proliferation of the MDS cell lines. After 48 h of co-culture MSC-exo inhibited differentiation of SKM-1 (*P* < 0.001) and MUTZ-1 cells (*P* < 0.01) and suppressed the expression of the proliferation marker CD117 (*P* < 0.001) in SKM-1 cells compared to the control, which was consistent with the results of the CCK-8 assay. Similarly, CD117, a cell proliferation marker, was not detected in MUTZ-1 cells (Fig. 7B, C). We found that MSC-derived exosomes increased the proportion of the G0/G1 phase cells in MDS cells (*P* < 0.001, *P* < 0.01) after 48 h of co-culture (Fig. 7D, E). We found that MSC-exo promoted apoptosis in MDS cells *(P* < 0.001) (Fig. 7F, G). Finally, we also examined the microenvironment alterations after 48 h of co-culturing. For SKM-1 cells, IL-2, IL-4, IL-6, IL-10, IL-17, TNF-α, and IFN-β were either significantly elevated or with elevation tendency. Interestingly, only IL-6 was significantly elevated in MUTZ-1 cells (*P* < 0.001, *P* < 0.01), while the rest of the cytokines did not differ significantly or even showed a decreasing trend (Fig. 7H).

## Discussion

One-third of MDS patients will transform into AML, and 24% of MDS patients have been reported to die as a result of leukemic transformation [27], However, the mechanisms of conversion are not yet fully understood and it is vital to examine the mechanisms of MDS to AML conversion and the treatment before conversion. Our study reveals for the first time the role of AML cell lines as well as MSC-derived exosomes in the conversion of MDS to AML.

We first extracted AML cell line-derived exosomes by ultrafiltration and then characterized them by transmission electron microscopy, nanoparticle tracking analysis, protein blotting, and flow cytometry. Next, AML cell line-derived exosomes were co-cultured with MDS cell lines, and exosomes were also labeled with PKH26 to demonstrate that the exosomes could be taken up by MDS cell lines and function as intercellular communication. After co-culturing for 48 h and detection of cell proliferation by CCK-8, we found that exosomes of AML cell line origin inhibited the growth of MDS cell lines, which we further verified with CD117, a protein closely related to proliferation, also known as c-kit [28], THP-1-exo inhibited the expression of CD117 in the SKM-1 cell line, while K562-exo did not affect the expression of CD117 in the SKM-1 cell line, probably because the co-culture time was not long enough to show any significant phenomenon. CD117 was not detected in the MUTZ-1 cell line, probably because the MUTZ-1 cell line does not express CD117. Cell cycle, differentiation, and apoptosis were analyzed by flow cytometry after 48 h of co-culture. Exosomes of AML cell line origin blocked the cell cycle of the SKM-1 cell line in the G0/G1 phase and the MUTZ-1 cell line in the S phase, and in general, exosomes of AML cell line origin blocked the cell cycle progression of the MDS cell line. In addition, AML cell line-derived exosomes promote the differentiation of MDS cell lines. Apoptosis assays have confirmed that AML cell line-derived exosomes promote apoptosis in MDS cell lines.

To further investigate how AML cell line-derived exosomes cause apoptosis in MDS cell lines, we again examined the alteration of seven cytokines and reactive oxygen species after 48 h co-culturing. We found that co-culturing resulted in more secretion of TNF-α and ROS from MDS cell lines. TNF-α is a key ligand in the death receptor signal pathway and ROS is an important signaling molecule in the mitochondrial pathway, both of which activate the Caspase protease family in a cascade reaction that ultimately activates Caspase3 protein to cause apoptosis [29]. We also verified the expression of downstream Caspase3 protein using protein blotting and the results were consistent with expectations.

Mesenchymal stem cells (MSC) are multipotent stem cells with the ability of self-renewal and multi-directional differentiation, which are widely distributed in many tissues of the human body, such as bone marrow, umbilical cord, and fat [30]. Some studies have found that bone marrow mesenchymal stem cells (BM-MSC) derived exosomes can induce apoptosis of leukemia cell lines, which may play a role in the treatment of leukemia [31]. Another study has carried out a detailed study on its specific mechanism, and suggested that it may be through hsa-miR-124-5p in BM-MSC derived exosomes to inhibit cell proliferation and cycle progression [32]. In this study, we used ultrafiltration to extract MSC- derived exosomes and co-cultured them with the MDS cell line for 48 h before performing the relevant assays. We found that MSC-derived exosomes significantly inhibited the proliferation of the MDS cell line, while CD117 expression was significantly reduced. In addition, MSC-derived exosomes inhibit the differentiation of MDS cell lines and promote apoptosis in MDS cell lines, while it blocks the MDS cell line cycle in the G0/G1 phase. The final cytokine results showed that MSC-derived exosomes can cause the release of several cytokines from MDS cell lines, with IL-6 showing a tendency to be elevated in both cell lines. It has been shown that IL-6 can act synergistically with other cytokines to promote the growth of early bone marrow stem cells and promote the development of blood cells [33, 34].

## Conclusion

**Fig. 8:**
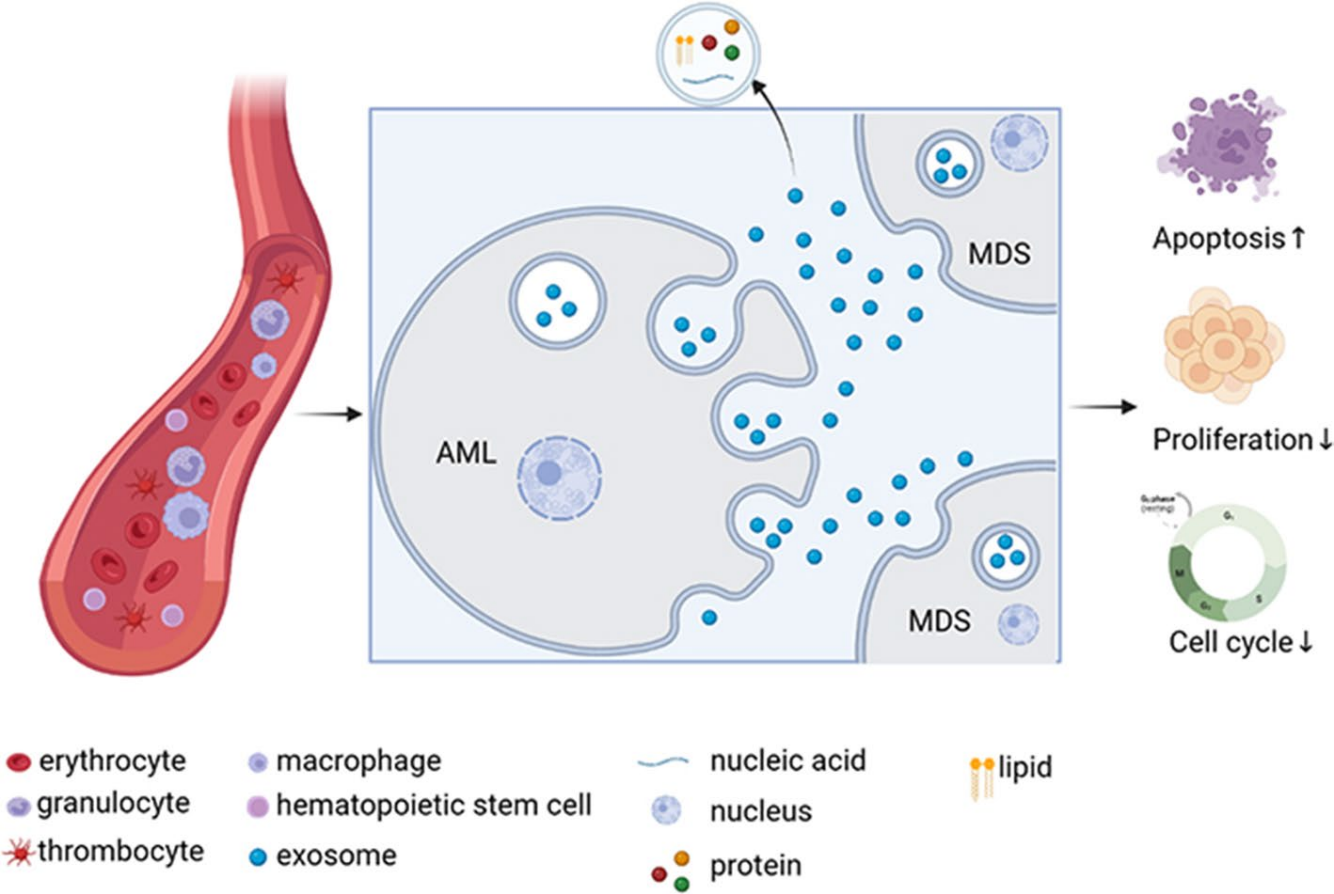
Transformation pattern of AML cell line-derived exosomes affecting MDS

In conclusion, our study shows that exosomes derived from AML cell lines can inhibit the proliferation of MDS cell lines, block cell cycle progression, promote apoptosis and differentiation, and lead to increased secretion of TNF-α and ROS in MDS cells. The data from this study suggests that AML-derived exosomes can affect the apoptosis of MDS cell lines via the TNF-α/ROS-Caspase3 pathway, thus affecting the transformation of MDS to AML (Fig. 8). We also found that MSC-derived exosomes inhibited the proliferation of MDS cells, blocked cell cycle progression, promoted apoptosis, inhibited cell differentiation, and released more IL-6. Whether this means that MSC-derived exosomes can play a role in the treatment of MDS patients needs further study.

## Data Availability

All data generated during this study are in this published article.
